# Axl and EGFR Dual-Specific Binding Affibody for Targeted Therapy in Nasopharyngeal Carcinoma

**DOI:** 10.3390/cells13221823

**Published:** 2024-11-05

**Authors:** Saidu Kamara, He Wen, Yanru Guo, Ying Liu, Lei Liu, Wangqi Du, Jun Chen, Shanli Zhu, Lifang Zhang

**Affiliations:** Institute of Molecular Virology and Immunology, Department of Microbiology and Immunology, School of Basic Medical Sciences, Wenzhou Medical University, Wenzhou 325035, China; 13676516320@163.com (S.K.); mildnotwild@163.com (H.W.); gyr0227@126.com (Y.G.); 18647509030@163.com (Y.L.); 17861170053@163.com (L.L.); dwq123654@126.com (W.D.); chenjun_8787@163.com (J.C.); wenzhouzsl@126.com (S.Z.)

**Keywords:** affibody molecules, nasopharyngeal carcinoma, Axl, EGFR, targeted therapy

## Abstract

Nasopharyngeal carcinoma (NPC) is a tumor of the head and neck, with a higher incidence in southern China and Southeast Asia. Radiotherapy and chemotherapy are the main treatments; however, metastasis and recurrence remain the main causes of treatment failure. Further, the majority of patients are diagnosed in the late stage due to lack of tumor-specific biomarker for early diagnosis. Therefore, an effective treatment and early detection can improve the outcome of patient with NPC. Axl and EGFR are co-expressed in NPC tissues and play key roles in tumor proliferation, migration, and invasion, which are often correlated with poor prognosis and therapy resistance. In this study, we generated a novel bispecific affibody (Z_239-1907_) for the dual targeting and inhibition of Axl and EGFR expression in NPC-positive cells both in vitro and in vivo. The in vitro experiments demonstrated that Z_239-1907_ had more pronounced antitumor effects than either modality alone (Z_AXL_239 or Z_EGFR_1907) in NPC-positive cells. Further, mice bearing NPC-positive tumors showed significant inhibition in tumor growth after treatment with Z_239-1907_ compared to Z_AXL_239 and Z_EGFR_1907. The in vivo tumor targeting ability and imaging also showed that Z_239-1907_ specifically and selectively targeted NPC xenograft mice models and accumulate at tumor site as early as 30 min and disappeared within 24 h post-injection. Collectively, these results suggest that Z_239-1907_ dual-target affibody is a promising therapeutic agent and a molecular imaging probe for early diagnosis in NPC.

## 1. Introduction

Over the last decade, immunotherapy has emerged as a hot spot in cancer treatment by providing a new and promising mode of therapy options for patients with solid tumors and hematologic malignancies [1]. Different types of cancer immunotherapy, including immune checkpoint inhibitors (ICIs) (anti-CTLA-4, anti-PD-1, and anti-PD-L1) and adoptive cell transfer (ACT) have shown durable clinical responses in a wide variety of tumors; however, patient benefit remains limited [2,3]. Further developments of new approaches are being explored to improve the outcome of cancer immunotherapy. One of the most promising strategies involves the combination of dual ICIs or the use of bispecific antibodies (bsAb) [4,5]. A recent study showed that inhibiting PD-1, PDL1, and CTLA-4 at the same time has achieved great success in non-small-cell lung cancer, urothelial carcinoma, and melanoma [6,7,8,9]. The development of bsAb can target two distinct antigens simultaneously with a wide range of applications, including redirecting T-cells against tumor cells, blocking two different signaling pathways, and the simultaneous targeting of multiple disease mediators [5,10]. As of recent years, the FDA has approved several bispecific antibodies for medical use, including blinatumomab, cadonilimab, tebentafusp, amivantamab, glofitamab, etc. [10,11]. Although many challenges are still faced, such as low response rate, tumor heterogeneity, treatment resistance, and systemic side effects, which hinder the applications of bsAbs. Gradually, with the recent advances in genetic engineering tools, as well as numerous biotechnological methods such as phage display, protein engineering, and transgenic animal technology, several formats of bsAb have been developed alongisde their application in cancer therapy [12]. These diverse formats circumvent previous challenges of bsAb, thus providing a wide range of options to tailor the design of bsAb to match the proposed mechanisms of action and the intended purpose and application. 

Epidermal growth factor receptor (EGFR) is a transmembrane glycoprotein that constitutes one of four members (EGFR, HER-3, HER-2/neu, and HER-4) of the ErbB family of tyrosine kinase receptors and consists of an extracellular ligand-binding domain, an intracellular region, and a hydrophobic transmembrane region [13,14]. The extracellular domain initiates ligand-receptor binding, which leads to activation in cytoplasmic domain and the phosphorylation of tyrosine receptor, which triggers downstream signaling pathways, such as PI3K/AKT, JAK/STAT, and Ras/Raf/MEK/ERK. These signaling pathways play pivotal roles in tumor cell proliferation, migration, invasion, and survival [15,16]. EGFR overexpression has been reported and implicated in the pathogenesis of many cancers, including nasopharyngeal carcinoma (NPC) [17]. It is reported that EGFR is highly expressed in about 80% of NPCs and is a poor prognostic factor [18,19]. Targeting EGFR therapies such as monoclonal antibodies and EGFR tyrosine kinase inhibitors (EGFR-TKIs) have been developed, which has helped clinicians to identify and treat patient with NPC [20]. However, toxicities and other adverse effects have been reported for several anti-EGFR therapies. Therefore, promising diagnostics and therapeutics are being developed to aid in the management of NPC.

The tyrosine kinase receptor Axl is a protein-coding gene and is a member of the Tyro3-Axl-Mer (TAM) receptor tyrosine kinase family [21]. Structurally, Axl includes an extracellular domain, a transmembrane domain, and an intracellular domain [22]. The extracellular domain consists of two immunoglobulin (Ig)-like domains and two fibronectin III domains involved in ligand binding [23]. The intracellular domain is crucial for autophosphorylation and also triggers subsequent kinase activity [24]. Axl binds the high-affinity ligand growth-arrest-specific protein 6 (GAS6), which has been implicated to promote cell survival, proliferation, migration, and invasion in many cancer types [25,26]. The overexpression of Axl has been identified in a variety of human malignancies such as breast cancer [27], nasopharyngeal carcinoma [28], melanoma [29], and prostate cancer [30]. Axl is highly expressed in NPC tissues and can promote the tumorigenesis and progression of NPC. A published study reported that Axl mRNA and protein expressions of NPC tissues were highly increased compared to those in normal nasopharyngeal epithelial tissues. Another similar study by Paccez et al. revealed that the expression of Axl was significantly correlated with metastasis to distant sites, and high-grade tumor–node–metastasis (TNM) classification, and also had poor overall survival in NPC patients [31]. Moreover, this is further supported by the facts that Axl promotes the expression of matrix metalloproteinase 9 (MMP-9), required for migration and invasion both in vitro and in vivo [32]. Furthermore, Axl was found to be highly expressed in prostate cancer with higher proliferation, migration, and invasion via the activation of PI3K/ NF-κB signaling pathways [33]. A recent study demonstrated that Axl small-molecule inhibitor hinders glioblastoma growth, migration, and invasion in vitro and in vivo [34], thus highlighting the need for targeting Axl specifically in NPC diagnosis and treatment.

Over the last three decades, monoclonal antibodies (mAbs) have been considered to be potent antitumor agents that have remarkably improved treatment outcomes because of their high degree of specificity [35]. However, mAbs also come with significant drawbacks for applied purposes, namely, their large molecular size, exhibition of limited distribution to solid tissues, poor thermal and chemical stability, and high cost of large manufacturing [36]. These limitations justified the need for engineered derivatives with smaller size and composition. In this context, other biological molecules with high-affinity binding properties toward ligands have been identified as powerful alternatives to mAbs. Several non-antibody protein scaffolds have proven to be an invaluable tool in diagnostics, therapy, and biotechnological applications. Among all, affibodies are one of the most important engineered protein scaffolds with a small size (6.5 kDa), high target affinity, and specificity capable of binding to a large variety of different protein targets [37]. HER-2 was one of the first tumor targets to be explored alongside the generation of a high-affinity HER-2 binding affibody molecule [38]. Interestingly, it was found that both monomer and dimeric affibodies had the same tumor uptake [39], suggesting that small size is of great importance. Furthermore, in a study by Gong et al., a labeled EGFR-specific affibody with a near-infrared (NIR) fluorescent probe was prepared, and in vivo imaging was performed in mice bearing EGFR-positive subcutaneous A431 tumors. The tumor could be detected in the first hour after injection, and images of dissected tissues confirmed high uptake in the tumor and liver [40]. To date, all reported bivalent affibodies have shown enhanced tumor-targeting selectivity and have uses in targeted cancer imaging [41,42]. However, to the best of our knowledge, no fused Axl-targeting affibody with an EGFR targeting affibody to form dual-target affibody for the diagnosis and targeted therapy of NPC in nude mice model has been reported.

In this study, we linked Z_AXL_239 and Z_EGFR_1907 monomers, with substantially improved binding affinity. Z_AXL_239-targeting AxL and Z_EGFR_1907-targeting EGFR were used since bispecific affibody showed better antitumor effects than monospecific affibody [43]. Moreover, Axl homo-dimerization, EGFR homo-dimerization, and Axl-EGFR heterophilic dimerization provide a gateway to pro-invasive signaling in cancer cells and acquired resistance to conventional therapy [44]. Therefore, it is of great importance to investigate the Axl-EGFR hetero-dimerization that initiates downstream signaling pathways involved in regulating cellular proliferation, differentiation, and survival, contributing to NPC tumorigenesis. 

## 2. Materials and Methods 

### 2.1. Materials

The vector *pET21a* (+) was purchased from Amersham Pharmacia Biotech and *Escherichia coli* (*E. coli*) BL21 from American Type Culture Collection (ATCC) Novagen. Reagents used included M13K07 helper phage (New England Biolabs, MA, USA), and *Sfi I*, *Not I*, *NdeI,* and *Xhol* restriction endonucleases were ligated together with a T4 Ligase (Thermo Fisher Scientific, Waltham, MA, USA), glutathione agarose affinity column, horseradish peroxidase- anti-M13 monoclonal antibodies (GE Healthcare, Uppsala, Sweden), Isopropyl β-D-1-thiogalactopyranoside (IPTG), paraformaldehyde and Triton X-100 (Sigma-Aldrich, Saint Louis, MO, USA), Ni-NTA agarose column, and bicinchoninic acid assay (BCA) kit (Beyotime, Beijing, China). Culture mediums Dulbecco’s Modified Eagle’s Medium (DMEM), Roswell Park Memorial Institute 1640 (RPMI-1640), fetal bovine serum (FBS), penicillin, and streptomycin were purchased from Gibco, Tokyo, Japan. Fluorescein isothiocyanate (FITC)-conjugated goat anti-mouse, IgG-antibody-conjugated goat anti-rabbit, Hoechst 33342, tetramethylbenzidine (TMB), and 5-Ethynyl-2′-deoxyuridine (EdU) were bought from Biotech (Beijing, China) Co., Ltd. Dylight 755 (Thermo Fisher Scientific, Waltham, MA, USA), cell lysis buffer (Beyotime, Beijing, China), Cell Counting Kit-8 (CCK-8; Dojindo, Kumamoto, Japan), Nuclear/Cytosol Extraction Kit (Applygen, Beijing, China) were also purchased. Antibodies were obtained from Cell Signaling Technology.

### 2.2. Construction of Affibody Combinatorial Library of the Z Domain 

A random phage display library was prepared [45], and a wild staphylococcal protein a-derived Z (SPA-Z) scaffold was used as sample for PCR amplification with primers encoding helices 1 and 2 of the Z domain. The gene fragments were digested with restriction endonuclease *Sfi I* and *Not I* (Thermo Fisher Scientific, Waltham, MA, USA), cloned into pCANTAB5E (Novagen and Amersham Pharmacia Biotech) to construct pCANTAB5E/SPA-N and expressed in *Escherichia coli* TG1. The affibody naïve library was cloned into vector that was found to contain about 1 × 10^9^ and had 100% diversity in the SPA-Z scaffold. Then, we evaluated the capacity and randomness of the inserted affibody library. After, the phage stocks were used to pan potential affibody that specifically bind to Axl (26-96 aa) using phage display technology.

### 2.3. Selection of Potential Binding Affibody to Axl 

Recombinant Axl protein with high purity was prepared and used as a target for the panning of phage display library. The phage selection for specific binders to Axl was performed by enzyme-linked immunosorbent assay (ELISA). As previously described [45], the library was subjected to three rounds of panning, and positive clones were identified by ELISA and sequenced. The phage clones with the high specific binding affinity to Axl were selected as potential affibody.

### 2.4. Construction of the Recombinant Plasmid

The amino acid sequences of two monomer affibodies Z_AXL_239 (generated in our laboratory) and Z_EGFR_1907 (generated by Friedman et al.) were linked with Ser/Gly linkers connecting various domains [46]. The His_6_-tagged Z_AXL239-EGFR1907_ product was digested with *NdeI* and *Xhol* and ligated to plasmid *pET21a* (+). The *pET21a* (+)/Z_AXL239-EGFR1907_ plasmid was transformed into competent *E. coli* cells using the calcium chloride method. After identification by sequencing, the recombinant plasmid was confirmed and designated as His_6_-tagged *pET21a* (+)/Z_239-1907_.

### 2.5. Protein Expression and Purification 

The Z_239-1907_ recombinant protein was induced by 1 mmol/L IPTG for 6 h. Supernatant of cell lysate was purified using Ni-NTA affinity column. The purified proteins were detected by SDS-PAGE and confirmed by Western blotting assay with anti-his-tag mouse monoclonal antibody. The BCA method was used to measure the protein concentrations, and then the purified proteins were aliquoted and stored at −80 °C until use.

### 2.6. Surface Plasmon Resonance (SPR)

Z_239-1907_, Z_AXL_239, and Z_EGFR_1907 binding to native AXL protein and native EGFR protein was assessed by BIAcore T200 SPR instrument (GE Healthcare, Uppsala, Sweden). AXL and EGFR target proteins were immobilized on the CM5 (GE Healthcare) BIAcore-sensor chip. Five different concentrations (10, 5, 2.5, 1.25, and 0.625 μM) were injected to flow over the immobilized BIAcore-sensor chip. The wild SPA-Z scaffold (Z_WT_) was set as negative control. Afterwards, the data were fitted to a 1:1 Langmuir model to determine the binding kinetic parameters using BIAcore T200 evaluation 3.0.2 software.

### 2.7. Cell Lines

NPC-positive cell lines C666-1, NPC/HK-1 (express both Axl and EGFR proteins), MKN-45 (express EGFR), and NPC-negative cell line TC-1 were all purchased from ATCC and used for evaluation of Z_239-1907_, Z_AXL_239, and Z_EGFR_1907 cell binding and inhibitory effects on cell proliferation in vitro. C666-1, NPC/HK-1 MKN-45, and TC-1 were cultured in either RPMI 1640 or DMEM media, supplemented with 10% FBS and antibiotics. Cells were maintained at 37 °C in humidified incubator in atmosphere containing 5% CO_2_. 

### 2.8. AXL and EGFR Expression Level in NPC-Positive Cell Lines

To examine the Axl and EGFR expression level in C666-1, NPC/HK-1, MKN-45, and TC-1 cell lines, Western blotting assay was employed. Cells were cultured for 48 h and then collected and lysed in lysis buffer. Cell lysate proteins were separated on a 10% SDS-PAGE gel and were transferred onto polyvinylidene difluoride membranes (Millipore, Burlington, MA, USA). Membranes were blocked with 5% skim milk in Tris-buffered saline (TBS) and then incubated with AXL and EGFR antibody overnight at 4 °C, followed by fluorescent antibody. The membrane was visualized using a fluorescence imaging system (Clinx, Shanghai, China. GADPH was used as internal control.

### 2.9. Immunofluorescence Assay

C666-1, HK-1 MKN-45, and TC-1 were plated on glass coverslips in a density of 1 × 10^3^ 6-well plates and maintained in a humidified incubator for 24 h at 37 °C. Then, cells were incubated with either Z_239-1907_, Z_AXL_239, Z_EGFR_1907, or Z_WT_ for 3 h at a concentration of 100 µg/mL. After, the cells were rinsed in PBS and then fixed, and permeabilized for 10 min (4% paraformaldehyde and 0.3% triton x-100). Cells were blocked with blocking buffer and incubated with the anti-his-tag mouse monoclonal primary antibody overnight at 4 °C. The cells were then incubated with FITC-conjugated goat anti-mouse secondary antibody. The cell nuclei were counterstained with Hoechst 33342 and imaged using a fluorescence microscope (Nikon C1-i, Tokyo, Japan).

### 2.10. In Vivo Tumor Targeting and Imaging

We investigated in vivo near-infrared imaging to assess Z_239-1907_, Z_AXL_239, and Z_EGFR_1907 specific tumor targeting ability in xenograft mice model. C666-1 and NPC/HK-1 cells (2 × 10^6^) were injected subcutaneously into the right forearm of nude mice (BALB/c, 4–5 weeks old, *n* = 3 per group). When tumor volume reached 300–500 mm^3^, mice were used for imaging. Z_239-1907_, Z_AXL_239, Z_EGFR_1907, and Z_WT_ were labeled with Dylight-755 (Thermo Fisher Scientific, Waltham, MA, USA) according to the manufacturer’s protocol. Mice were then injected with 100 µg of Dylight-755-labeled affibody dissolved in PBS (150 µL) in the tail vein, and images were obtained at different time points using the NIR imaging system (Cri Maestro 2.10, Cambridge Research & Instrumentation, Inc., Cambridge, MA, USA). The fluorescence intensity of tumor-to-skin ratios were measured 0, 4, and 24 h after tail vein injection.

### 2.11. In Vitro Inhibitory Effects 

Cell viability assays were performed to determine the inhibitory effect of Z_239-1907_, Z_AXL_239, and Z_EGFR_1907 on the proliferation of C666-1, NPC/HK-1, MKN-45, and TC-1 cells. Cells were seeded at a density of 5 × 10^3^ cells/well into 96-well plates. After 24 h of incubation, cells were treated with increasing concentration of affibody ranging from 0.625 μM to 20 μM. The cell viability was determined after 0, 12, 24, 36, 48, and 72 h incubation with either Z_239-1907_, Z_AXL_239, or Z_EGFR_1907. Then, 10 μL of the CCK-8 solution was added into each well and incubated for 30 min. The absorbance was determined using microplate reader at a wavelength of 450 nm (OD value).

### 2.12. 5-Ethynyl-2′-deoxyuridine Cell Proliferation Assay

Cell proliferation was measured by EdU staining (Beyotime, Beijing, China). In brief, C666-1, NPC/HK-1, MKN-45, and TC-1 cells were seeded in a density 2.5 × 10^4^ cells per well and then treated with 10 μM Z_239-1907_, Z_AXL_239, Z_EGFR_1907, or Z_WT_ for 24 h at 37 °C. Next, EdU labeling medium was added to the culture and incubated for 2 h at 37 °C. After, cells were fixed and stained with Hoechst 33342. Three random microscopic fields were imaged and the positive cells were counted. Magnification, 400×.

### 2.13. Wound Healing Assay

Briefly, C666-1, NPC/HK-1, MKN-45, and TC-1 cells were seeded in six-well plate at 1 × 10^5^ cells/well. After cells grew to confluence, a 10 µL pipette tip was used to create a wound across the cell monolayer. The cells were rinsed with PBS and then cultured in serum-free RPMI-1640 medium containing 10 μM Z_239-1907_, Z_AXL_239, Z_EGFR_1907 or Z_WT._ The degrees of wound closing were observed under a light microscope at 0, 24, and 48 h after wound creation. Magnification, 100×.

### 2.14. Western Blotting Analysis

C666-1 and NPC/HK-1 cells were seeded in six-well plate at 1 × 10^5^ cells/well. Then, cells were incubated with either Z_239-1907_, Z_AXL_239, Z_EGFR_1907, or Z_WT_ for 36 h at 37 °C. Total protein was extracted using RIPA lysis buffer (Beyotime, Shanghai, China), and protein concentration was measured using BCA protein assay kit. The extracted proteins were separated by 10% SDS-PAGE and transferred to PVDF membrane. Thereafter, the PVDF membrane was blocked with 5% skim milk and incubated with the appropriate primary antibodies, followed by incubation with the appropriate secondary antibodies. Protein bands were visualized by Western blotting imaging system, and GADPH was used as internal control.

### 2.15. In Vivo Therapeutic Efficacy 

After establishment of C666-1 and NPC/HK-1 tumor-bearing mice models, the tumor-bearing mice were divided into 5 groups at random and injected intravenously with Z_239-1907_, Z_AXL_239, Z_EGFR_1907, and Z_WT_ 100 nmol/kg every three days for 30 days. Positive control cisplatin was given at a dose of 5 mg/kg. Negative control PBS was given at a dose of 100 µL, respectively. Then, we further evaluated the effectiveness of different treatment regimens by daily measurements of body weight and tumor size. Tumor-bearing mice were sacrificed after 30 days of treatment, and the implanted tumors were removed to be photographed, weighed and stored at −80 °C.

### 2.16. Statistical Analysis

Data are presented as the mean ± standard deviation (SD). Statistical analysis of the results was performed using Student’s test. A *p* value <0.05 was regarded as statistically significant. Graph pad prism software was used for all data analysis.

## 3. Results

### 3.1. Expression, Purification, and Identification of Affibody Molecules

**Figure 1:**
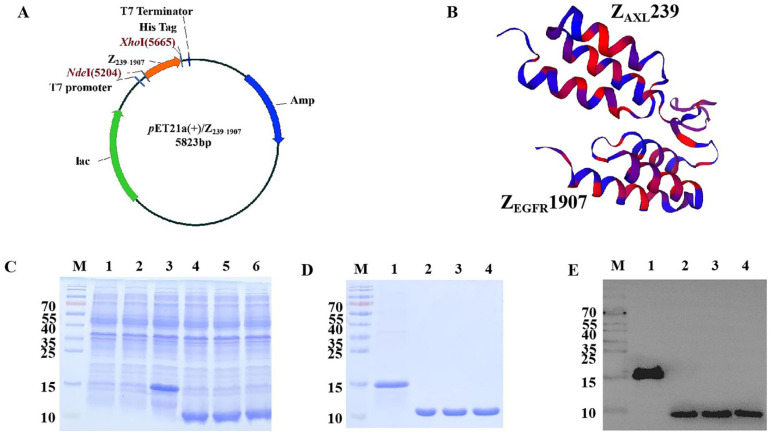
**Protein expression and purification**. (**A**) Schematic representation of *pET21a* (+)/Z_239-1907_ plasmid construct. (**B**) Three-dimensional structure prediction by Swiss model. (**C**) SDS-PAGE and Coomassie brilliant blue staining of the recombinant proteins. M, protein marker; 1, empty *E. coli* BL21; 2, transformed *pET21a* (+) empty vector of *E. coli* BL21; 3–6, *E. coli* BL21 transformed with *pET21a* (+)/Z_239-1907_, *pET21a* (+)/Z_AXL_239, *pET21a* (+)/Z_EGFR_1907, and *pET21a* (+)/Z_WT_ induced with IPTG, respectively. (**D**) Purified proteins analyzed by SDS-PAGE. (**E**) Protein identification was further confirmed by Western blot.

After three rounds of panning, positive phage clones were identified by ELISA and sequenced. One hundred clones were randomly selected for sequencing, resulting in a total of 63 high-quality sequences. Four different sequences with higher OD450 values were selected as potential Z_AXL_ affibodies. Among them, Z_AXL_239 was used and linked with Z_EGFR_1907 to obtain Z_239-1907_ dual-target affibody. The Z_239-1907_ nucleic acid sequence was inserted into *pET21a* (+) plasmid and expressed in *E. coli* as a His-tagged protein (Figure 1A). Predicting the three-dimensional (3-D) structure from amino acid sequence (Figure 1B). The plasmid was transformed into *E. coli* BL21, and protein expression was induced by IPTG (Figure 1C). The expressed protein was then purified by Ni-NTA affinity chromatography (Figure 1D), and the identity of the expressed protein was confirmed by Western blot (Figure 1E). 

### 3.2. Binding Affinity of Affibody Molecules to Target Proteins

**Figure 2:**
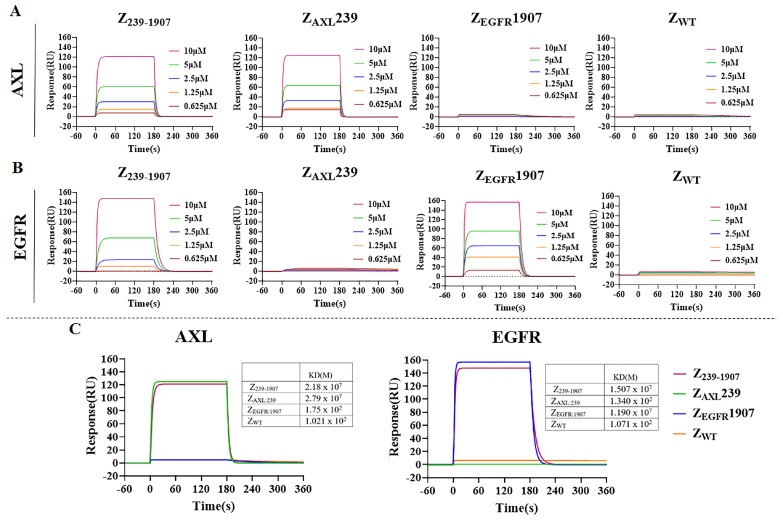
**Binding analysis**. (**A**,**B**) Sensograms measuring the binding interaction of Z_239-1907_, Z_AXL_239, Z_EGFR_1907, and Z_WT_ to Axl and EGFR, respectively. (**C**) Sensograms obtained after injection of the highest concentration 10 μM of affibodies to flow over the sensor chips. Z_WT_ was used as a negative control. Three independent experiments were performed in triplicate.

Surface plasmon resonance (SPR) was used to determine the binding affinity of Z_239-1907_ bispecific affibody to Axl and EGFR. The dual-target and monospecific affibodies were injected at different concentrations over flow-cell surfaces of a sensor chip containing immobilized Axl and EGFR recombinant proteins. The results demonstrated that Z_239-1907_ dual-target affibody is detectable by SPR in a concentration-dependent manner and can simultaneously bind to both Axl and EGFR, whereas the monospecific affibodies Z_AXL_239 and Z_EGFR_1907 only bind to their respective targets (Figure 2A,B). As expected, the negative control affibody SPA-Z scaffold (Z_WT_) did not show any binding to Axl and EGFR. Then, we measured the dissociation equilibrium constant (KD) values of Z_239-1907_, Z_AXL_239, Z_EGFR_1907, and Z_WT_ to Axl are 2.19 × 10^7^ mol/L, 2.79 × 10^7^ mol/L, 1.75 × 10^2^ mol/L, and 1.02 × 10^2^ mol/L, and the KD values of Z_239-1907_, Z_AXL_239, Z_EGFR_1907, and Z_WT_ to EGFR are 1.51 × 10^7^ mol/L, 1.34 × 10^2^ mol/L, 1.19 × 10^7^ mol/L, and 1.07 × 10^2^ mol/L, respectively. Our data confirmed that monospecific and dual-target affibodies have similar binding affinities; however, Z_239-1907_ dual-target affibody bind to both Axl and EGFR compared to Z_AXL_239 and Z_EGFR_1907.

### 3.3. In Vitro Cellular Binding of Affibody Molecules

**Figure 3:**
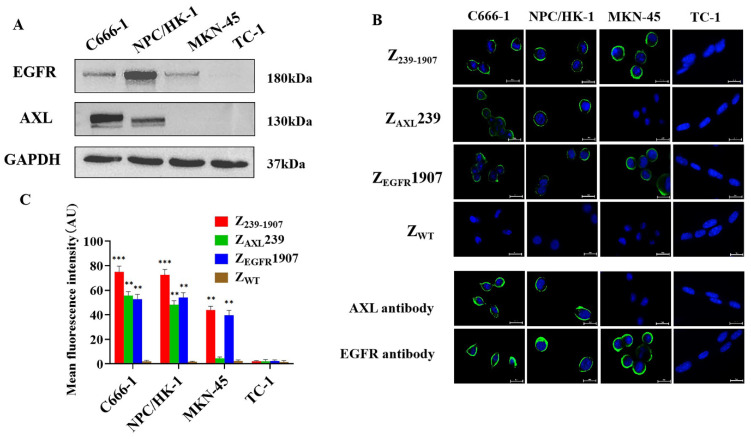
**Immunofluorescence assay**. (**A**) Western blot protein expression of NPC-positive cell lines and NPC-negative cell line. (**B**) C666-1, NPC/HK-1, MKN-45, and TC-1 cell lines were treated with Z_239-1907_, Z_AXL_239, Z_EGFR_1907, or Z_WT_. Corresponding antibodies are labeled with FITC (green), and cell nuclei are stained with Hoechst 3342 (400×). (**C**) Mean fluorescence intensity of Z_239-1907_, Z_AXL_239, Z_EGFR_1907, and Z_WT_-treated cell lines. Data are given as mean ± SD (*n* = 3). ** *p* < 0.01; *** *p* < 0.001 compared to SPA-Z scaffold (ZWT).

We firstly investigated the expression level of Axl and EGFR in NPC-positive and NPC-negative cell lines by Western blotting. As shown in (Figure 3A), C666-1, NPC/HK-1, and MKN-45 cell lines displayed substantial Axl and EGFR expression; however, the TC-1 cell line did not show the expression of Axl and EGFR. Next, we investigated the cellular binding specificity of the affibody molecules to NPC-positive tumor cells over-expressing Axl and EGFR using an indirect immunofluorescence assay. As shown in Figure 3B, a bright-green fluorescence signal appeared on the cell membrane of C666-1, NPC/HK-1, and MKN-45, which were treated with Z_239-1907_, Z_AXL_239, or Z_EGFR_1907 affibody molecules, as well as strong fluorescence signals in cells treated with Z_239-1907_ than Z_AXL_239 and Z_EGFR_1907 (Figure 3C). Nevertheless, C666-1, NPC/HK-1, and MKN cells treated with Z_WT_ and TC-1 treated with Z_239-1907_, Z_AXL_239, or Z_EGFR_1907 did not show obvious green fluorescence signals. Axl and EGFR antibodies were used as positive controls. These results suggest that Z_239-1907_ have higher binding affinity in cells where Axl and EGFR are expressed compared to Z_AXL_239 and Z_EGFR_1907.

### 3.4. In Vivo Tumor Targeting and Imaging of Affibody Molecules

**Figure 4:**
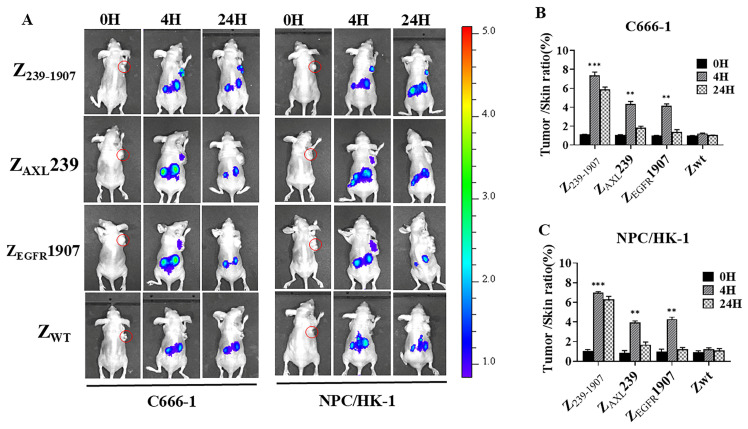
**NIR fluorescence tumor imaging.** (**A**) Images were recorded at different time points after injection with Dylight-755-labeled Z_239-1907_, Dylight-755-labeled Z_AXL_239, or Dylight-755-labeled Z_EGFR_1907. Dylight-755-labeled Z_WT_ was set as negative control. (**B**,**C**) Calculated tumor/skin fluorescence intensity ratio post-injection of affibody molecules. Data are given as the mean ± SD (*n* = 3). ** *p* < 0.01; *** *p* < 0.001 compared to SPA-Z scaffold (Z_WT_).

In nude mice bearing C666-1 and NPC/HK-1 cell lines, Dylight-755-labeled Z_239-1907_, Dylight-755-labeled Z_AXL_239, and Dylight-755-labeled Z_EGFR_1907 were quickly distributed throughout the body 1 h after injection, peaked at 4 h, and were rapidly cleared from the nude mice within 24 h. The NIR fluorescence images were obtained at different time points after the tail vein injection of Dylight-755-labeled Z_239-1907_, Dylight-755-labeled Z_AXL_239, Dylight-755-labeled Z_EGFR_1907, and Dylight-755-labeled Z_WT_ (Figure 4A). The tumor/skin fluorescence intensity ratio post-injection of affibody molecules can be observed in Figure 4B,C. The Dylight-755-labeled Z_WT_ did not show fluorescence signals in nude mice bearing a tumor-cell xenograft. Taken together, our results further proved that tumors in mice that were injected with Dylight-755-labeled Z_239-1907_ displayed higher fluorescence intensity than tumors in mice injected with Dylight-755-labeled Z_AXL_239 and Dylight-755-labeled Z_EGFR_1907. 

### 3.5. In Vitro Inhibitory Efficacy of Affibody Molecules

**Figure 5:**
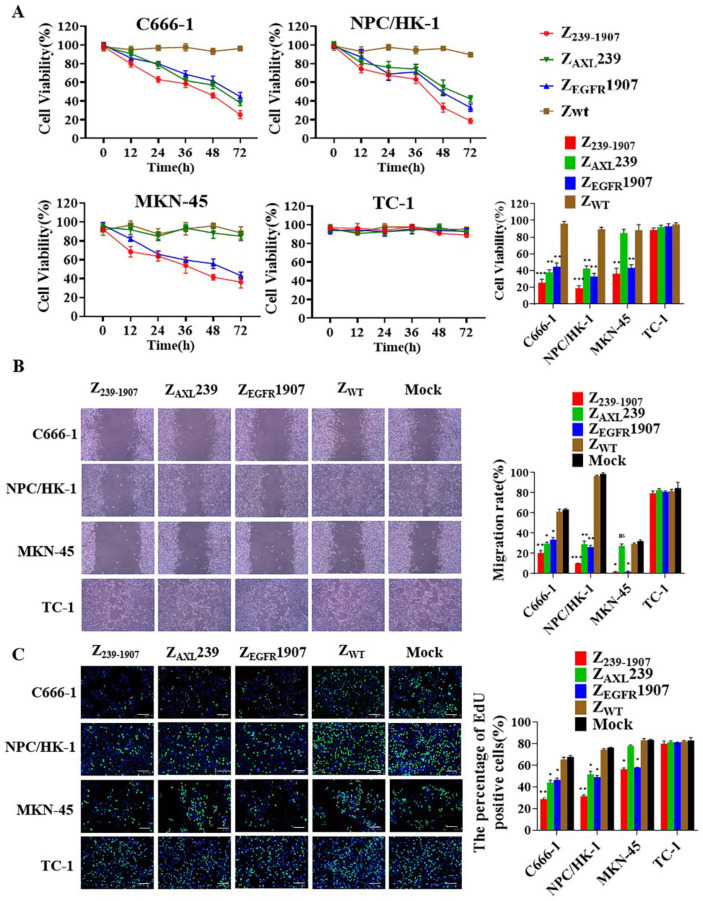
**Affibody molecules inhibited NPC-positive cell proliferation.** (**A**–**C**) CCK-8, wound-healing, and EdU were used to determine the effect of Z_239-1907_, Z_AXL_239, and Z_EGFR_1907 on the proliferation of NPC-positive cells. Z_WT_ set as negative control. Data are given as the mean ± SD (*n* = 3). * *p* < 0.05; ** *p* < 0.01; *** *p* < 0.001 compared to SPA-Z scaffold (Z_WT_).

We performed CCK-8, wound-healing, and EdU assays to examine the effect of affibody molecules on the proliferation of NPC-positive cells. Firstly, cells were treated with an increasing concentration of affibody molecules for a time period. After treatment with either Z_239-1907_, Z_AXL_239, or Z_EGFR_1907 affibody molecules, the cell viability was found to decrease in a dose-dependent manner (Supplementary Figure S1). We next investigated the potency of Z_239-1907_, Z_AXL_239, and Z_EGFR_1907 affibody molecules over the course of 0, 12, 24, 36, 48, and 72 h. In C666-1 and NPC/HK-1, Z_239-1907_ displayed higher inhibitory efficacy than Z_AXL_239 and Z_EGFR_1907 but in MKN-45 cell Z_239-1907_ and Z_EGFR_1907 had similar inhibitory effects. However, Z_AXL_239 had no inhibitory effect in the MKN-45 cell. Furthermore, Z_239-1907_, Z_EGFR_1907, and Z_EGFR_1907 affibody molecules did not show any inhibitory effects in the TC-1 cell. As expected, Z_WT_ had no effects in the cell lines used (Figure 5A). Next, we employed wound-healing assay to study the effects of affibody molecules on the migration of NPC cells. As shown in Figure 5B and Figure S2, cells treated with Z_239-1907_ significantly reduced the rate of migration compared to Z_AXL_239 and Z_EGFR_1907; however, the TC-1 cell treated with Z_239-1907_, Z_AXL_239, or Z_EGFR_1907 did not alter the speed of cell migration.

To further investigate the effects of these affibody molecules on the proliferation of NPC-positive cells, EdU was performed. As shown in Figure 5C, EdU staining was greatly reduced in NPC-positive cells in which DNA replication was blocked by treatment with Z_239-1907_ compared to Z_AXL_239 and Z_EGFR_1907. Nevertheless, the TC-1 cell treated with Z_239-1907_, Z_AXL_239, or Z_EGFR_1907 did not inhibit cell proliferation. These results confirmed our hypothesis that Z_239-1907_ showed superior efficacy over Z_AXL_239 and Z_EGFR_1907 alone in NPC-positive cells.

### 3.6. Affibody Molecules Treatment Blocked Signaling Pathways Stimulated by Axl and EGFR

**Figure 6:**
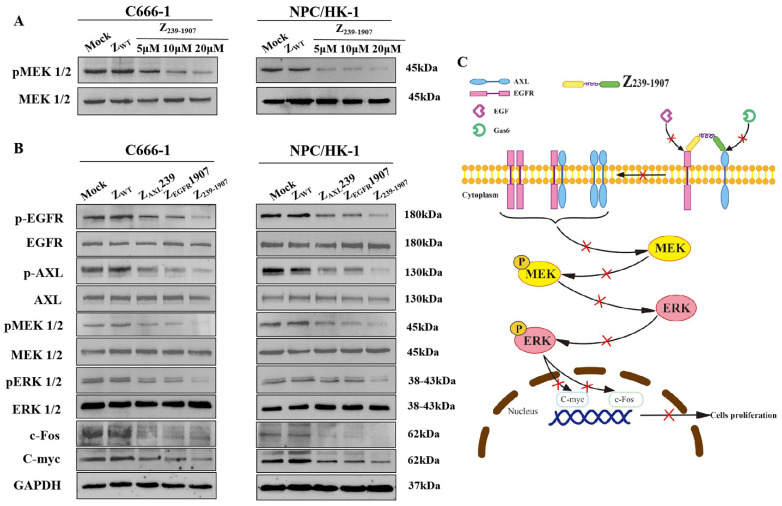
**Signal transduction pathway**. (**A**) Downregulation of p-MEK after Z_239-1907_ treatment in a concentration- and time-dependent manner. (**B**) Treatment with either Z_239-1907_, Z_AXL_239, or Z_EGFR_1907 downregulated signaling proteins and transcription genes. (**C**) Image of the schematic representation of Z_239-1907_ affibody molecule blocking MAPK-ERK signaling pathway. Experiments were performed in triplicate.

Both Axl and EGFR have been identified as key players in NPC tumor cell invasiveness. However, the mechanisms underlying the downstream signaling of Axl and EGFR in NPC have not yet been completely understood. This study investigated the MAPK-ERK pathway with emphasis on its dysregulation in NPC-positive cell lines, as well as using affibody molecules to target MAPK-ERK pathway. The p-MEK was significantly downregulated after Z_239-1907_ treatment in a concentration- and time-dependent manner compared to the mock and Z_WT_ group (Figure 6A). As shown in Figure 6B, the oncogenic signaling molecules p-EGFR, p-Axl, p-MEK, and p-ERK were downregulated after treatment with either Z_239-1907_, Z_AXL_239, or Z_EGFR_1907 in C666-1 and NPC/HK-1 cells, as well as the downregulation of c-Fos and c-Myc genes transcription. Altogether, our results confirmed that the Z_239-1907_ dual-target affibody had better effects on the MAPK-ERK pathway than Z_AXL_239 and Z_GFR_1907. 

### 3.7. In Vivo Antitumor Efficacy of Affibody Molecules in NPC Xenograft Nude Mouse Model

**Figure 7:**
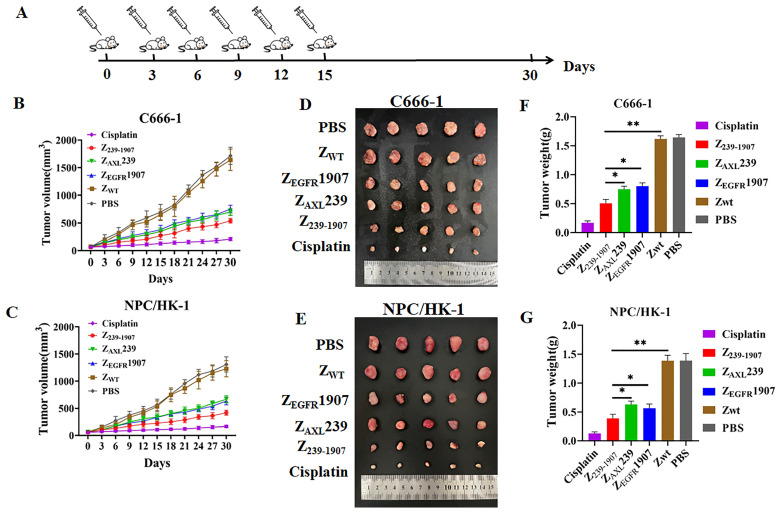
**Assessment of therapeutic efficacy of affibody molecules.** (**A**) Schematic illustration of affibody molecules treatment in mouse model. (**B**,**C**) Tumor volume of mice with different treatments (PBS, Z_WT_, Z_239-1907_, Z_AXL_239, Z_EGFR_1907, and cisplatin, n= 6/group). (**D**,**E**) Tumor separated from mice under different treatment regimens. (**F**,**G**) Tumor weights in different groups of mice. Data are given as mean ± SD (*n* = 5). * *p* < 0.05; ** *p* < 0.01 compared to SPA-Z scaffold (Z_WT_).

The in vivo antitumor efficacy of affibody molecules was evaluated in C666-1 and NPC/HK-1 bearing nude mice model by measuring the tumor growth of the mice. As shown in Figure 7B–E, tumors in saline-treated mice and Z_WT_-treated mice grew much faster and larger in size, whereas mice treated with Z_239-1907_, Z_AXL_239, Z_EGFR_1907, or cisplatin decreased tumor growth. Tumor weight in a different group of mice can be observed in Figure 7F,G. These results further proved that the administration of Z_239-1907_ significantly reduced tumor growth after 15 days of treatment. This decrease was higher than that of Z_AXL_239 and Z_EGFR_1907-treated mice but was lower than that observed in cisplatin-treated mice.

## 4. Discussion

The genetic alteration in cancer cells or the abnormal expression of signaling components disrupts the regulatory nodes that control cell function, enabling cells to undergo de-regulated mitogenesis, protects from apoptosis, and provides the ability to invade surrounding tissues [47]. Therefore, the development of novel therapies that target multiple oncogenic signaling pathways is critical as therapies targeting single-target biomarker or pathway have shown limited efficacy. Recently, several bifunctional multi-targeting small molecules have been developed for the treatment of malignancies [48]. In this study, we discuss Z_239-1907_ affibody simultaneously targeting Axl and EGFR and the therapeutic efficacy of Z_239-1907_ in NPC-positive cells both in vitro and in vivo.

With the development of new treatment strategies, NPC has seen improved treatment outcomes as a result in advances in radiotherapy, chemotherapy, and immunotherapy [49]. However, these regimens are often associated with significant toxicity and limited efficacy [50]. Therefore, the use of a small-molecule-targeted anti-tumor drug has the potential to revolutionize NPC patient outcome. Currently, anti-Axl and anti-EGFR therapies (mAbs and TKIs) have demonstrated significant clinical benefits for the treatment of patient with NPC; however, acquired resistance to Axl or EGFR in tumor cells is a major obstacle in NPC management [51,52]. In this regard, simultaneous targeting increases therapeutic efficacy in cancer patients when compared to mono-therapeutic approaches [53]. For example, bispecific antibodies targeting EGFR and receptor tyrosine kinases (RTKs) have shown better clinical therapeutic effects than monospecific antibodies in cancer treatment [54]. Further, EGFR-targeted antibodies and antibody-drug conjugates have been shown to enhance treatment efficacy against tumors with EGFR expression [54]. In this present study, we generated Z_239-1907_ for dual targeting and the inhibition of Axl and EGFR expression in NPC-positive cell lines. Our in vitro experiments (CCK-8, wound-healing, and EdU assays) showed that Z_239-1907_ had more pronounced antitumor effects than that obtained in either monospecific affibody (Z_AXL_239 or Z_EGFR_1907) on NPC-positive cell lines but had no antitumor effects towards the NPC-negative cell line. Furthermore, mice bearing NPC-positive tumors showed significant inhibition in tumor growth after treatment with dual-target affibody compared to the monospecific affibodies. Overall, these results proved that Z_239-1907_ dual-target affibody was far superior to Z_AXL_239 and Z_EGFR_1907 because Z_239-1907_ displayed better antitumor effects in NPC-positive cells both in vitro and in vivo, and also no obvious toxic effects were observed in the NPC-negative cell.

The aberrant activation of intracellular signaling pathways are known to exert critical functions in human cancer cells, influencing the tumor microenvironment and promoting cancer development and progression [55]. The Axl-EGFR heterophilic dimerization provides a gateway to pro-invasive signaling and limits response to EGFR- and AXL-targeted inhibitors in NPC cells [44]. However, the underlying mechanism of how Axl and EGFR crosstalk downstream signaling has not yet been completely understood. The aim of the present study was to evaluate bispecific affibody inhibitory effects on downstream signaling proteins of the Axl and EGFR pathway in NPC cells. Our results showed that Z_239-1907_ inhibited tumor cell growth, which blocked the activation of the Axl–EGFR signaling pathway in NPC cells. Further, Z_239-1907_ also inhibited the expression of the proto-oncogenes c-Fos and c-Myc. Taken together, our results showed that Z_239-1907_ has the capacity to limit NPC-positive cell proliferation, suggesting therapeutic potential for patients with NPC.

Molecular imaging is an emerging discipline that allows visualization of intra-tumor regions, improving the early-stage diagnosis of malignant tumors, and plays a crucial role in targeted therapy [56]. It relies on imaging probes for the detection of disease-specific biomarkers [57]. To date, antibodies have been extensively utilized in clinical practice, mainly because of their high target specificity and binding affinity. They are perfect candidates for the molecular imaging of disease diagnosis and prognosis evaluation. Nevertheless, there are also concerns associated mainly with the large size of antibodies, poor tumor penetration, and unspecific accumulation, which causes a poor signal-to-background ratio that reduces imaging contrast [58,59,60,61]. On the other hand, various protein scaffold molecules with a smaller size, better tumor penetration, and shorter clearance time have shown advantages and much promise in cancer imaging diagnosis [62]. To explore the efficacy and specificity of tumor-targeting ligand for NPC-positive cells, we developed Z_239-1907_ affibody shown to possess high-binding affinity for Axl and EGFR. The Z_239-1907_ affibody was approved to bind to both Axl and EGFR, which are highly expressed in NPC tissues. The in vivo tumor targeting ability and imaging showed that Z_239-1907_ specifically targeted NPC xenograft mice models, which was consistent with our in vitro results. Moreover, the accumulation of Dylight-755-labeled Z_239-1907_ at the tumor site was achieved as early as 30 min and disappeared within 24 h post-injection, similar to published studies such as affibody-based nanoprobes specifically targeting HER-2 and affibody and affitoxin that target EBV-LMP2 [40,62]. These results strongly suggest that Z_239-1907_ affibody has high binding specificity for NPC-positive cells expressing Axl and EGFR and the potential for clinical use as molecular imaging probe for the early diagnosis of NPC.

In summary, the in vitro and vivo studies revealed that Z_239-1907_ had better antitumor effects than either Z_AXL_239 or Z_EGFR_1907 in NPC-positive cell lines. In addition, Z_239-1907_ bispecific affibody displayed excellent tumor-targeting ability in NPC-positive tumor and has great application potential for the in vivo molecular imaging diagnosis of NPC. Overall, these results suggest that Z_239-1907_ dual-target affibody may be promising for Axl-EGFR targeted therapy and molecular probe for tumor imaging in NPC. 

## Data Availability

The original contributions presented in the study are included in the article/Supplementary Material. Further inquiries can be directed to the corresponding author.

## Supplementary Materials

The following supporting information can be downloaded at https://www.mdpi.com/article/10.3390/cells13221823/s1. Supplementary Figure S1: C666-1, NPC/HK-1 and MKN-45 cell lines were treated with different concentrations of Z_239-1907_, Z_AXL_ 239, and Z_EGFR_ 1907 for 72 h. IC50 values were calculated using graph pad prism. Supplementary Figure S2: cells treated with Z_239-1907_ for 24 h significantly reduced the rate of migration compared to Z_AXL_239 and Z_EGFR_1907. However, TC-1 cell treated with Z_239-1907_, Z_AXL_239 or Z_EGFR_1907 did not alter the speed of cell migration.
