# Supplementary material for: Axl and EGFR Dual-Specific Binding Affibody for Targeted Therapy in Nasopharyngeal Carcinoma
Source: Cells. 2024 Nov 5;13(22):1823. doi: 10.3390/cells13221823 (PMC11592995; doi:10.3390/cells13221823)

Supplementary materials

Journal:

Cells

Title:

AXL and EGFR Dual Specific Binding Affibody for Targeted Therapy in Nasopharyngeal Carcinoma

Authors:

Saidu Kamara*, He Wen *, Yanru Guo, Ying Liu, Lei Liu, Wangqi Du, Jun Chen, Shanli Zhu, Lifang Zhang

* These authors contributed equally to this work.

Institute of Molecular Virology and Immunology, Department of Microbiology and Immunology, School of Basic Medical Sciences, Wenzhou Medical University, Wenzhou 325035, Zhejiang, PR China.

Corresponding author: Lifang Zhang (wmuzhanglifang@wmu.edu.cn)

Supplementary Figure S1. C666-1, NPC/HK-1 and MKN-45 cell lines were treated with different concentrations of $Z_{239\text{-}1907}$, Z_{AXL} 239, and Z_{EGFR} 1907 for 72 h. IC50 values were calculated using graph pad prism.

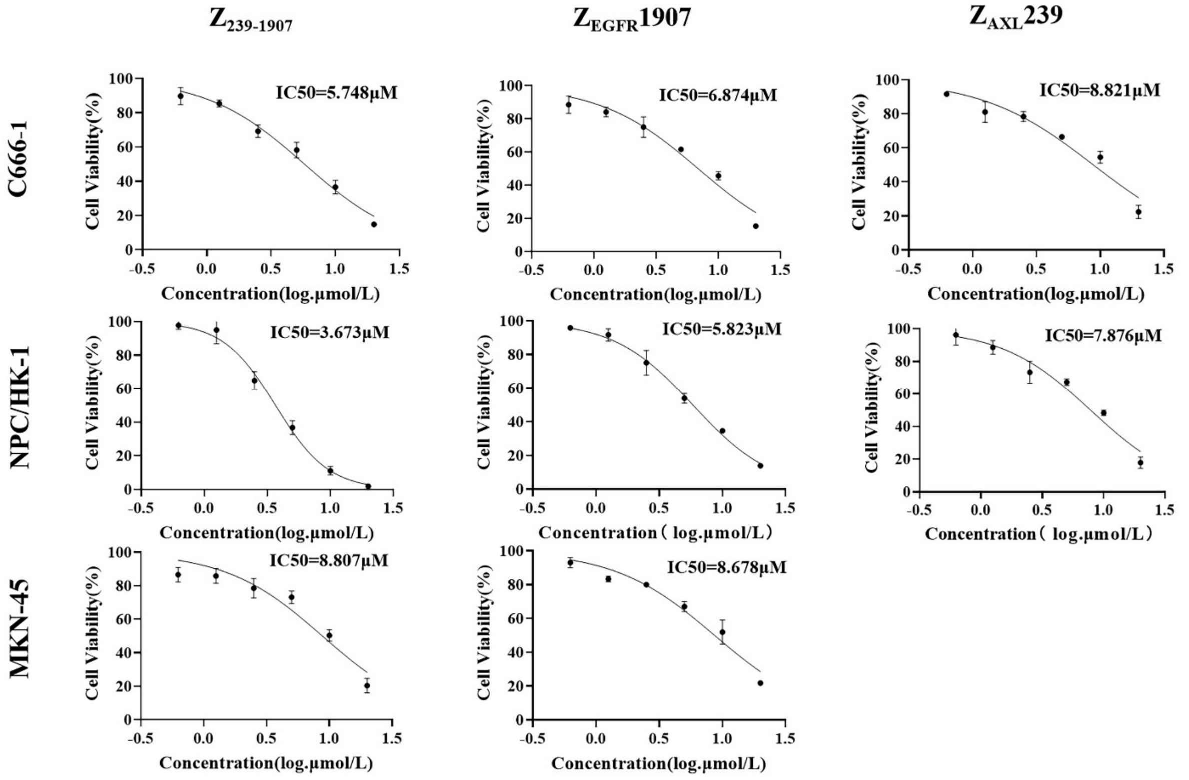

Supplementary Figure S2, cells treated with $Z_{239\text{-}1907}$ for 24 h significantly reduced the rate of migration compared to $Z_{AXL}239$ and $Z_{EGFR}1907$. However, TC-1 cell treated with $Z_{239\text{-}1907}$, $Z_{AXL}239$ or $Z_{EGFR}1907$ did not alter the speed of cell migration.

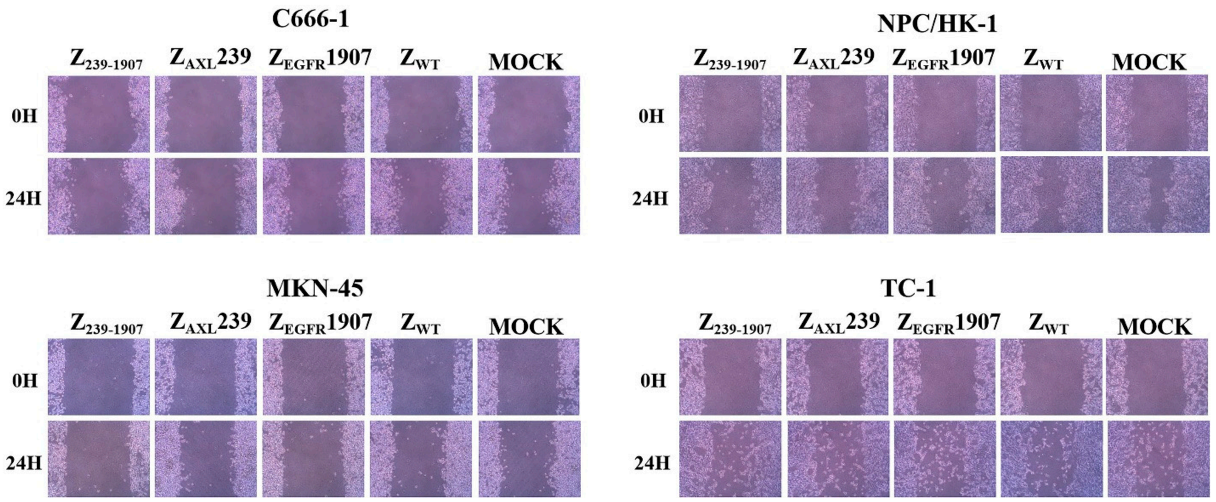

Supplement: Supplementary file 1 [file cells-13-01823-s001.zip › cells-3239778-supplementary.pdf]
